# *Lafoensia pacari* extract induces apoptosis mediated by caspase-3 and inhibition of growth in human lung cancer cells

**DOI:** 10.1186/1753-6561-7-S2-P70

**Published:** 2013-04-04

**Authors:** Yonara G Cordeiro, Arina L Rochetti, Antônio M Scatolini, Edson R Silva, Vinicius C Souza, Heidge Fukumasu

**Affiliations:** 1 Department of Basic Science, FZEA, University of Sao Paulo, Pirassununga, Brazil; 2 Department of Biological Science, ESALQ, University of Sao Paulo, Piracicaba, Brazil

## Background

*Lafoensia pacari* is a native species from South America which has been used in traditional medicine as anti-ulcerogenic and anti-inflammatory for several diseases, but the antineoplastic potential still have not been elucidated so far, though its etnopharmacological indication. The aim of this study was to evaluate the anti-neoplastic effect of *L. pacari* ethanolic extract in three human lung neoplastic cell lines.

## Materials and methods

For the assessment of cytotoxicity, cell lines were grown *in vitro*, being two originally from non-small cell lung carcinoma (A549 and H2023) and one of giant cell lung carcinoma obtained from pleural effusion (H460). Cells were grown in 96 well plates under regular cell culture condition and treated with different concentrations of *L.pacari* ethanolic extract, then analyzed using 3-(4,5-dimethylthiazol-2yl)-2,5-diphenyl bromide tetrazolium, widely used to determine the viability of cultured cells. The IC50 value and the regression curve were calculated with 5.0 Prism (GraphPad Software, USA). Also, the Caspase-3 Assay Kit for Live Cells (Biotium, USA) was used to determine the mode of action of *L. pacari* at IC50 concentration.

## Results

After the treatment for 72h with the ethanolic extract of *L.pacari*, we determined a dose-dependent effect of *L. pacari* extract in all cell lineages, being the H460 cell line the most sensitive by means of lowest IC50. Thus, we also showed that this effect was due to induced caspase-3 dependent apoptosis.

## Conclusions

The extract of *L. pacari* demonstrated an antineoplastic effect in all cell lines. The Caspase-3 activation in tumor cells after treatment with *L. pacari* suggests that the cytotoxic effect is related to activation of the intrinsic apoptotic pathway, leading ultimately to cell death. Further studies are under investigation to determine the specific substances responsible for these effects.

## Financial support

CAPES and FAPESP (2008/56584-2).

